# Retraction: Mortality Risk Factors for Patients With Sepsis-Induced Blood Pressure Drop: A Single-Center Retrospective Study

**DOI:** 10.7759/cureus.r49

**Published:** 2022-03-17

**Authors:** Vicky Kumar, Sabeen Sharif Khan, Yousef Awad, Zahoor Ur Reham, Fares Mohammed Saeed Muthanna, Ellen Huang, Marina Basta, Hajra Khwaja, Rahil Barkat

**Affiliations:** 1 Internal Medicine, South City Hospital, Irbid, JOR; 2 Medicine, Aga Khan University Hospital, Karachi, PAK; 3 Clinical Research, The Oncology Institute of Hope and Innovation, Cerritos, USA; 4 Internal Medicine, Al Rahba Hospital, Abu Dhabi, ARE; 5 Pharmaceutical Care, Walailak University, Nakhon Si Thammarat, THA; 6 Internal Medicine, Oceania University of Medicine, Apia, WSM; 7 School of Medicine, St. George's University, True Blue, GRD; 8 Research Department, Aga Khan University Hospital, Karachi, PAK; 9 Indus Hospital Research Center, The Indus Hospital, Karachi, PAK

This article has been retracted due to the unknown origin of the data, lack of verified IRB approval, and purchased authorships. The primary author, Rahil Barkat, was involved in data theft and misuse in two recently published Cureus articles, which have since been retracted.

As the origin of this article’s data and verified IRB approval cannot be confirmed, we have made the decision to retract this article. Cureus has confirmed that the co-authors were asked by Mr. Barkat to proofread the article and provide payment in exchange for authorship. (Proofreading is an insufficient contribution to warrant authorship as defined by ICMJE.) These payments were made in the guise of “editing fees” but greatly exceed any editing fees paid to Cureus. While these authors may have been defrauded by Mr. Barkat, they remain complicit due to their lack of honest contributions to the article.

